# High-resolution mapping of QTL for fatty acid composition in soybean using specific-locus amplified fragment sequencing

**DOI:** 10.1007/s00122-017-2902-8

**Published:** 2017-04-07

**Authors:** Bin Li, Shengxü Fan, Fukuan Yu, Ying Chen, Shengrui Zhang, Fenxia Han, Shurong Yan, Lianzheng Wang, Junming Sun

**Affiliations:** The National Key Facility for Crop Gene Resources and Genetic Improvement, NFCRI, MOA Key Laboratory of Soybean Biology (Beijing), Institute of Crop Science, Chinese Academy of Agricultural Sciences, 12 Zhongguancun South Street, Beijing, 100081 China

## Abstract

*****Key message***:**

**We constructed a high-density linkage map comprising 3541 markers developed by specific-locus amplified fragment sequencing, and identified 26 stable QTL including nine novel loci, for fatty acid composition in soybean.**

**Abstract:**

Soybean oil quality and stability are mainly determined by the fatty acid composition of the seed. In the present study, we constructed a high-density genetic linkage map using 200 recombinant inbred lines derived from a cross between cultivated soybean varieties Luheidou2 and Nanhuizao, and SNP markers developed by specific-locus amplified fragment sequencing (SLAF-seq). This map comprises 3541 markers on 20 linkage groups and spans a genetic distance of 2534.42 cM, with an average distance of 0.72 cM between adjacent markers. Inclusive composite interval mapping revealed 26 stable QTL for five fatty acids, explaining 0.4–37.0% of the phenotypic variance for individual fatty acids across environments. Of these QTL, nine are novel loci (*qLA1*, *qLNA2_1*, *qPA4_1*, *qLA4_1*, *qPA6_1*, *qSA12_1*, *qPA16_1*, *qOA18_1*, and *qFA19_1*). These stable QTL harbor three fatty acid biosynthesis genes (*GmFabG*, *GmACP*, and *GmFAD8*), and 66 genes encoding lipid-related transcription factors. These stable QTL and tightly linked SNP markers can be used for marker-assisted selection in soybean breeding programs.

**Electronic supplementary material:**

The online version of this article (doi:10.1007/s00122-017-2902-8) contains supplementary material, which is available to authorized users.

## Introduction

Soybean (*Glycine max* L. Merr.) is one of the most important oilseed crops worldwide. Providing most of the world’s supply of vegetable protein and oil, soybean accounted for approximately 61% of the world’s oilseed production in 2015 (http://soystats.com). The quality and stability of soybean oil are mainly determined by five predominant fatty acids, viz. palmitic, stearic, oleic, linoleic, and linolenic acids (Lee et al. 2007). Palmitic (16:0) and stearic (18:0) acids are saturated fatty acids, while oleic (18:1), linoleic (18:2), and linolenic (18:3) acids are unsaturated fatty acids. A high proportion of unsaturated fatty acids in the human diet benefits cardiovascular health (Connor 2000; Mensink and Katan 1992). However, polyunsaturated fatty acids, particularly linolenic acid, increase the oxidation of food oils, causing an off-flavor and reducing the shelf life of the oil (Hu et al. 1997; Mounts et al. 1988). Therefore, one important focus of soybean breeding is to improve the fatty acid composition in seed oil.

Fatty acid content is a quantitative trait that depends on the combined effects of several major and minor genes (Bilyeu et al. 2005; Fan et al. 2015; Wang et al. 2014). Therefore, quantitative trait loci (QTL) mapping is an effective method to uncover the genetic basis of fatty acid formation. To date, numerous QTL for fatty acid contents have been detected (Alrefai et al. 1995; Bachlava et al. 2009; Brummer et al. 1997; Diers and Shoemaker 1992; Fan et al. 2015; Hyten et al. 2004; Panthee et al. 2006; Reinprecht et al. 2006; Wang et al. 2012, 2014; Xie et al. 2012). However, these QTL span fairly large genomic regions due to the relatively low density of genetic maps. The relatively low accuracy of QTL mapping using these maps limits not only the identification of fatty acid biosynthesis and regulatory networks, but also the application of these QTL in marker-assisted selection (MAS) breeding efforts in soybean. Recently, putative nucleotide polymorphisms responsible for fatty acid contents, which are usually denoted as quantitative trait nucleotides (QTN), were also identified in genome-wide association study (GWAS) based on population-wide linkage disequilibrium (LD) using soybean natural populations and genome-wide single nucleotide polymorphisms (SNP) (Li et al. 2015). The annotated candidate genes bearing these QTN demonstrated that fatty acid formation is governed by a complex genetic basis in soybean (Li et al. 2015).

With the great development in next-generation sequencing (NGS), several procedures were developed for SNP discovery and genotyping in large population, including restriction-site associated DNA tag sequencing (RAD-seq), genotyping-by-sequencing (GBS), and specific-locus amplified fragment sequencing (SLAF-seq), etc. (Baird et al. 2008; Elshire et al. 2011; Sun et al. 2013). These procedures reduced the genome complexity by digesting genomic DNA with restriction enzymes, and the resultant reduced representation library (RRL) was sequenced to achieve SNP discovery and genotyping in large population. Specifically, a pre-design experiment was performed in SLAF-seq to evaluate restriction enzymes and sizes of restriction fragments using the soybean reference genome sequence, which improved the efficiency of SLAF-seq (Sun et al. 2013). Additionally, the fragments in GBS library were usually selected through PCR amplification (Elshire et al. 2011). In contrast, the fragments in SLAF library were gel-purified, and the fragments with specific size were selected in subsequent sequencing. That will improve the uniformity of fragments in RRL library (Sun et al. 2013). Previously, we developed 200 recombinant inbred lines (RILs) from a cross between two cultivated soybean with different fatty acid compositions. Using 100 of these 200 RILs, We constructed a linkage map consisting of 161 SSR markers, and the QTL for fatty acid composition were identified across 3 years (2009 through 2011) (Fan et al. 2015). In addition, we sequenced 110 of the 200 RILs and the two parents, and developed a high-density genetic map comprising 5785 markers based on the SLAF-seq method (Li et al. 2014).

In the current study, we developed a great number of SNP-based markers using SLAF-seq with an increased mapping population size of 200 RILs to improve the efficiency and accuracy of QTL mapping. The results will benefit the improvement of the fatty acid composition of soybean in breeding project.

## Materials and methods

### Plant materials and field trials

Two hundred F_5:7_ RILs developed from the Luheidou2 (LHD2)/Nanhuizao (NHZ) cross together with the parental lines were planted with three replicates in randomized complete blocks at Shunyi Experimental Stations (N40°13′ and E116°34′) in Beijing from 2009 to 2011. Each plot comprised a 2-m row, with 0.5 m apart between rows and a space of 0.1 m between adjacent plants (Fan et al. 2015). Both of the parents are wild type cultivated soybean varieties with black seed coats, but their fatty acid composition differs significantly (Fan et al. 2015).

### Fatty acid extraction and determination

The composition of five predominant fatty acids (palmitic, stearic, oleic, linoleic, and linolenic acids) was determined using gas chromatography (Fan et al. 2015). Briefly, 20 g of soybean seeds of each line were ground to a fine powder with a Sample Preparation Mill (Retsch ZM100, Φ = 1.0 mm, Rheinische, Germany). Three hundred milligrams of each powdered sample was transferred to a 2-ml centrifuge tube preloaded with 1.5 ml *n*-hexane. After vigorous mixing, the mixture was stored at 4 °C for 12 h. Then the samples were centrifuged at 5000×*g* (room temperature) for 10 min. The supernatant was collected and sodium methoxide solution was added. The mixture was shaken for 1 h on a twist mixer (TM-300, ASONE, Japan) for full methyl esterification of the fatty acids, and centrifuged again at 5000×*g* for 10 min. The supernatant was collected to determine the composition of the five fatty acids (Fan et al. 2015).

Fatty acid composition was determined using an RTX-Wax Column (30 m × 0.25 mm × 0.25 mm) of gas chromatography (GC-2010, SHIMADZU, Japan). The injection volume was 1 μL. Nitrogen, hydrogen and air were used as carrier gases. The temperature was initially set at 180 °C for 1.5 min, increased to 210 °C at a rate of 10 °C min^−1^, and maintained at 210 °C for 2 min, increased to 220 °C at a rate of 5 °C min^−1^ and maintained at 220 °C for 5 min. The area normalization method was used to calculate the composition (percentage of total fatty acids by mass) of the five fatty acids using a GC2010 workstation (Fan et al. 2015).

### Specific-locus amplified fragments (SLAF) library construction and sequencing

We previously constructed and sequenced a SLAF library for 110 of the 200 RILs (Li et al. 2014). In our current study, the SLAF library of the remaining 90 RILs and parents was constructed and sequenced, following the same method, with minor modifications: first, the genomic DNA of each sample was digested with a single restriction enzyme, *Mse*I, rather than both *Eco*RI and *Mse*I according to the pre-design experiment results based on the latest version of the soybean reference genome sequence (Wm82.a2.v1, https://phytozome.jgi.doe.gov) (Schmutz et al. 2010); second, amplified fragments that were 374–474 bp in length instead of 500–550 bp were gel-purified and diluted for pair-end sequencing using an Illumina high-throughput sequencing platform (Illumina, Inc; San Diego, CA, USA).

### SLAF-seq data grouping and genotyping

The SLAF-seq data grouping and genotyping of the 90 RILs were performed following the previously reported method (Li et al. 2014; Sun et al. 2013). Briefly, low-quality reads (quality score <20e) were filtered out and then raw reads were sorted to each progeny according to duplex barcode sequences using SLAF_Poly.pl software (Biomarker, Beijing, China). After the barcodes and the terminal 5-bp positions were trimmed from each high-quality reads, clean reads from the same sample were mapped onto the soybean reference genome sequence (Wm82.a2.v1) using SOAP software (Li et al. 2008b; Schmutz et al. 2010). Sequences mapping to the same position with over 95% identity were defined as one SLAF locus. Since soybean is a diploid species and one locus can only contain at most four SLAF tags, groups containing more than four tags were filtered out as repetitive SLAFs, and the SLAFs with 2–4 tags were identified as polymorphic SLAFs (Sun et al. 2013).

Genotype scoring was then performed using a Bayesian approach to further ensure the genotyping quality (Sun et al. 2013). First, a posteriori conditional probability was calculated using the coverage of each allele and the number of single nucleotide polymorphisms. Then, genotyping quality score translated from the probability was used to select qualified markers for subsequent analysis. Low-quality markers for each marker and each individual were counted and the worse markers or individuals were deleted during the dynamic process. When the average genotype quality scores of all SLAF markers reached the cutoff value, the process stopped. The resultant polymorphic SLAFs were integrated with those of other 110 RILs described in our previous study (Li et al. 2014), and the polymorphic SLAF markers for 200 RILs were obtained. Finally, high-quality SLAF markers for the genetic mapping were filtered by the following criteria: First, average sequence depths should >2-fold in each progeny and >10-fold in the parents. Second, markers with more than 25% missing data were filtered. Third, the Chi-square test was performed to examine the segregation distortion. Markers with significant segregation distortion (*P* < 0.05) were excluded. The final SNP-based polymorphic SLAF markers were used to construct a high-density linkage map.

### Construction of a high-density genetic map

Based on the genotyping data of 200 RILs, a high-density genetic map comprising 20 linkage groups (LGs) was constructed using the Kosambi mapping function of the Joinmap v4.0 software with a LOD threshold of 5.0. The collinearity of 20 LGs with the soybean reference genome was analyzed by plotting the genetic positions of SLAF markers against their physical positions in the soybean reference genome (Wm82.a2.v1).

### QTL mapping for fatty acid composition

The additive QTL for palmitic, stearic, oleic, linoleic, and linolenic acids were detected using inclusive composite interval mapping (ICIM) in the BIP (bi-parental populations) model of QTL IciMapping software v4.0 (Li et al. 2008a), with the *P* values for entering variables (PIN) = 0.05. The threshold of the logarithm of the odds (LOD) scores for evaluating the statistical significance of QTL effects was determined using 1000 permutations at the significance level of 0.05. As a result, a LOD score of 3.3 was used as the threshold to declare the presence of a QTL. As the fatty acid composition is affected by the environments, we focused mainly on the QTL for individual fatty acids identified across multiple environments. The epistatic effects of QTL were identified by the ICIM-EPI method based on the BIP model implemented in QTL IciMapping software v4.0, with PIN = 0.05. The LOD threshold of 5.0 was obtained through 1000 permutation to declare the epistatic QTL at the significance level of 0.05.

### Annotation of genes within additive QTL intervals

The sequences within QTL intervals were identified according to the soybean reference genome sequence (Wm82.a2.v1, https://phytozome.jgi.doe.gov), and annotated against Nr (non-redundant), Swiss-Prot, and KOG/COG (clusters of orthologous groups) databases using Blastx program (https://blast.ncbi.nlm.nih.gov).

## Results

### Phenotypic analysis of fatty acid compositions in soybean RIL population

The fatty acid compositions of 200 RILs were determined from 2009 to 2011. As shown in Table 1, the five predominant fatty acids exhibit broad ranges in 200 RILs. Of them, linoleic acid shows the minimum of coefficient of variance (CV) ranging from 4.3 to 6.9%, while stearic acid presents the maximum of CV ranging from 11.3 to 17.8%. The broad sense heritability of five predominant fatty acids ranged from 0.74 to 0.88 over 3 years, suggesting the fatty acids are mainly controlled by genetic factor (Table 1).

**Table 1:** The characteristics of five fatty acids in 200 soybean RILs from 2009 to 2011

Trait	Environment	Mean ± SD (%)	Minimum (%)	Maximum (%)	CV^a^	Variance	*P*_(K–S)_^b^	*H*_B_^2c^	*H*_BC_^2d^
Pamitic	2009	10.24 ± 0.57	8.78	13.25	0.056	0.33	0.563	0.81	0.75
	2010	10.89 ± 0.54	9.29	13.75	0.049	0.29	0.568	0.76	
	2011	10.75 ± 0.68	9.23	12.96	0.064	0.47	0.500	0.57	
Stearic	2009	3.84 ± 0.48	2.57	5.11	0.124	0.23	0.099	0.87	0.74
	2010	3.75 ± 0.43	2.62	4.95	0.113	0.18	0.968	0.77	
	2011	3.68 ± 0.66	2.22	7.06	0.178	0.43	0.515	0.63	
Oleic	2009	24.06 ± 2.67	19.61	35.07	0.111	7.11	0.216	0.88	0.81
	2010	26.47 ± 4.39	17.71	39.13	0.166	19.24	0.369	0.87	
	2011	22.26 ± 3.05	17.67	36.28	0.137	9.29	0.001	0.86	
Linoleic	2009	53.27 ± 2.31	45.95	57.50	0.043	5.32	0.744	0.88	0.80
	2010	51.40 ± 3.53	41.64	59.00	0.069	12.44	0.190	0.87	
	2011	55.05 ± 2.51	43.64	59.46	0.046	6.28	0.003	0.69	
Linolenic	2009	8.58 ± 0.69	6.72	10.56	0.081	0.48	0.830	0.78	0.88
	2010	7.48 ± 0.88	5.47	10.57	0.117	0.77	0.910	0.84	
	2011	8.26 ± 0.80	5.74	10.05	0.097	0.65	0.825	0.89	

^a^Coefficient of variance

^b^ *P* value in Kolmogorov–Smirnov test

^c^Broad-sense heritability in each environment

^d^Broad-sense heritability in combined environments (2009, 2010, and 2011)

**Fig. 1:**
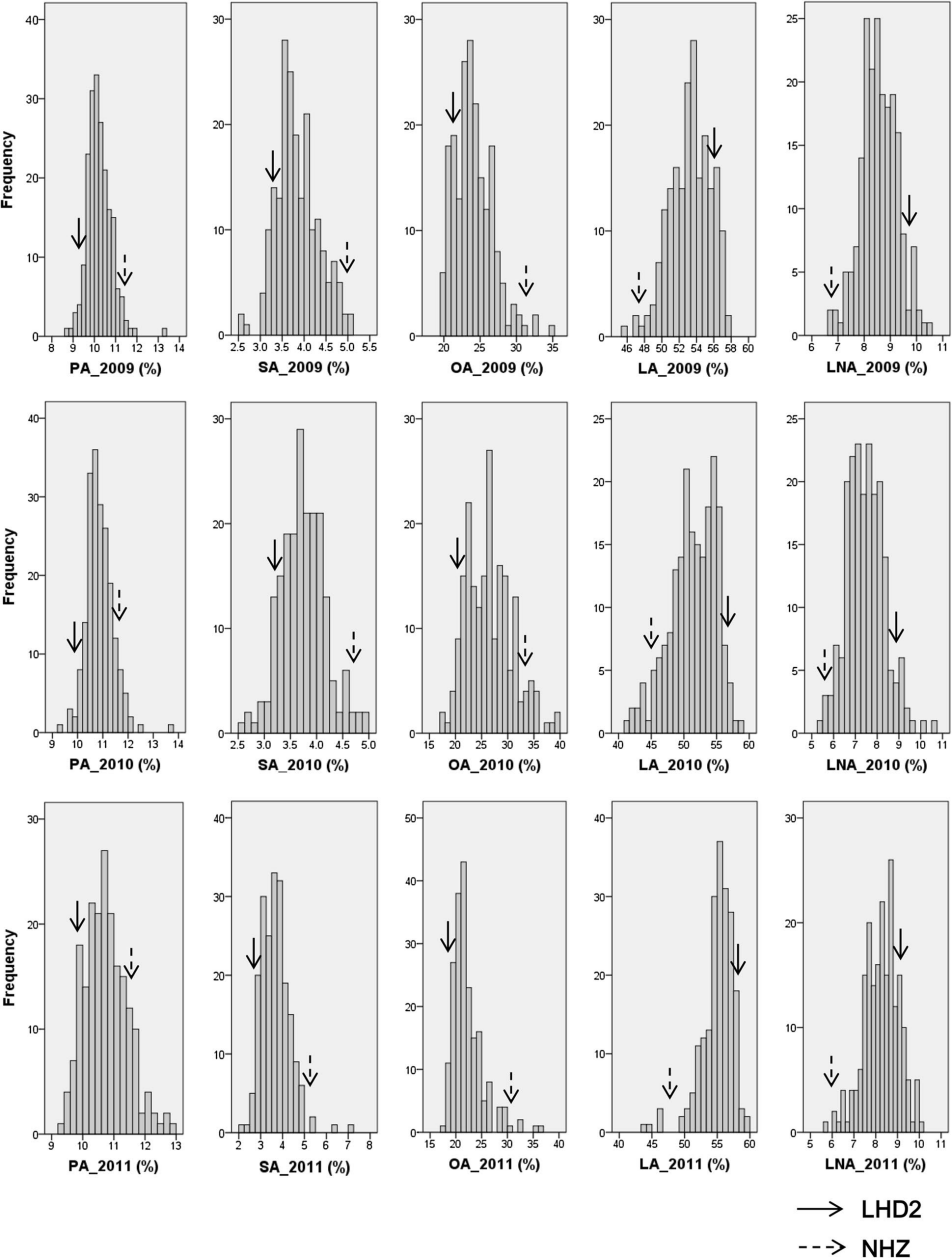
Frequency distributions of five fatty acid contents in 200 RIL seeds from 2009 to 2011. The *arrows* indicate the fatty acid compositions in two parental lines (cv. LHD2 and NHZ)

The frequency distributions of the five fatty acids were also analyzed. Almost all fatty acids exhibit continuous and normal distributions from 2009 to 2011, except oleic and linoleic acids in 2011 (Fig. 1; Table 1), suggesting fatty acids are inherited in a quantitative manner. Moreover, significant transgression segregations were also observed in progenies (Fig. 1), suggesting both parents contributed to fatty acid composition.

### SLAF-seq and genotyping of soybean RIL population

A total of 281.5 million pair-end reads from SLAF-seq were generated using the Illumina Genome Analyzer IIx in this study. Specifically, 28.4 and 28.9 million reads were generated for female parent LHD2 and male parent NHZ, respectively, while 224.3 million reads were obtained for 90 of the 200 RILs. After filtering, paired-end reads with clear information were mapped to the soybean reference genome (Version of Wm82.a2.v1, https://phytozome.jgi.doe.gov), and 453,524 effective SLAFs were developed. Polymorphisms of the integrated SLAFs were analyzed, and 16,199 polymorphic SLAFs were identified. These polymorphic SLAFs were integrated with the 9948 polymorphic SLAFs identified in other 110 RILs of the population in our previous study (Li et al. 2014), and integrated SLAFs were further screened to filter out markers that were unsuitable for genetic map construction. Finally, 3541 polymorphic SLAFs were obtained to construct a high-density linkage map. All of these markers are of the SNP-type.

### Construction of a high-density genetic map in soybean

**Fig. 2:**
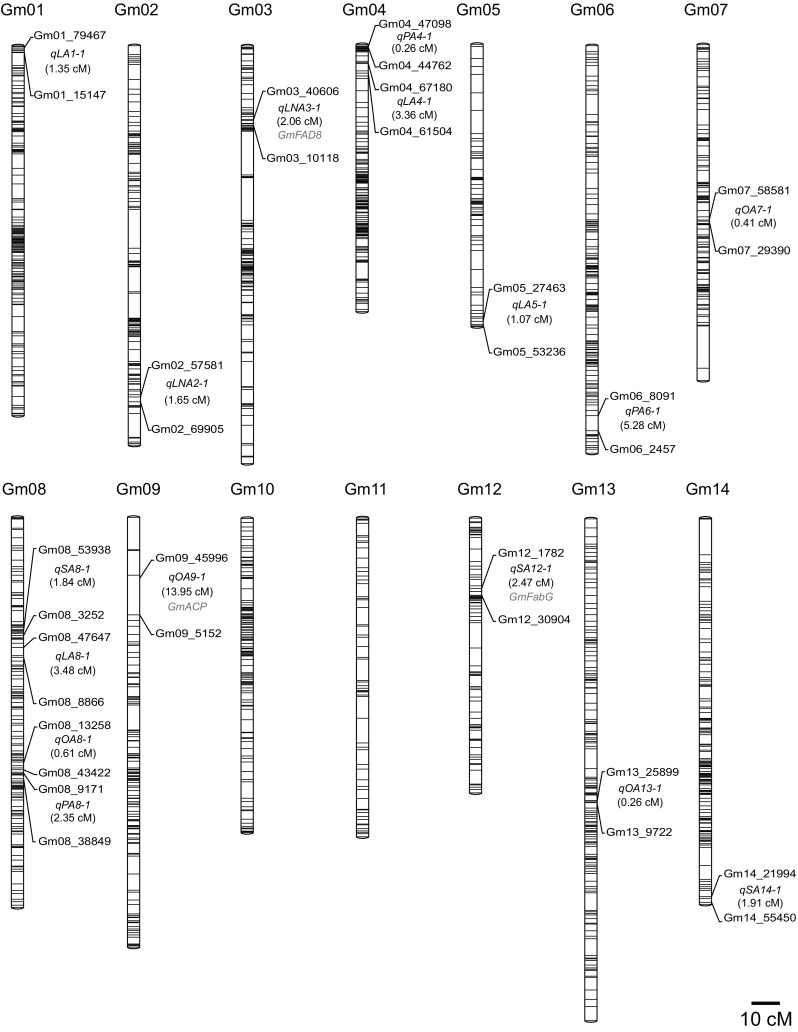

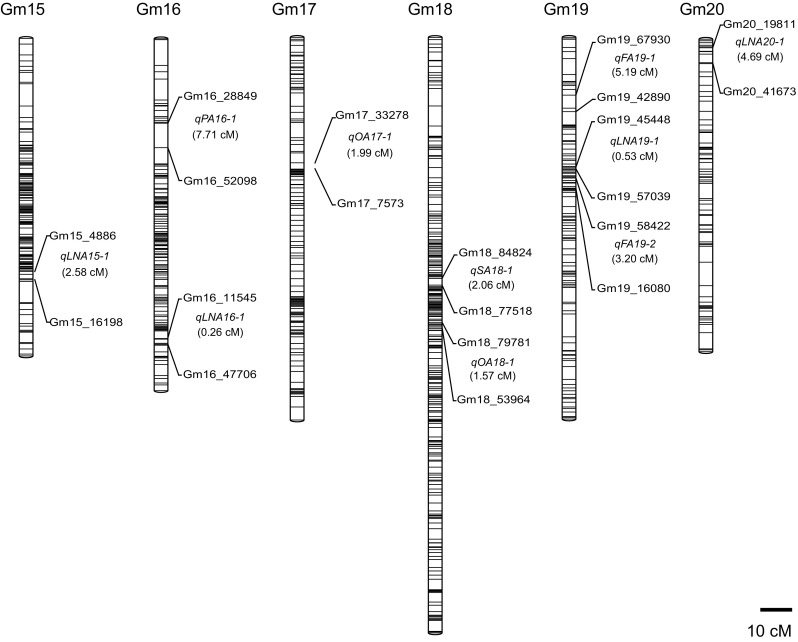
High-density genetic map for soybean and 26 stable QTL for fatty acids. The SLAF marker distributions were depicted on the 20 linkage groups (Gm01–Gm20) based on their genetic positions in centiMorgans (cM). The 26 stable QTL for fatty acids, along with their interval distances (cM) are shown between the tightly linked SLAF markers on the *right* side of each linkage group. The three fatty acid biosynthesis genes are indicated within the QTL intervals in *red* color by comparing their physical positions with that of SLAF markers in the soybean reference genome (color figure online)

The genotyping data of the 3541 SLAF markers for 200 RILs were analyzed to determine the order of these SLAF markers in 20 LGs, and a new high-density genetic map was constructed, with a genetic distance of 2534.42 cM (Fig. 2; Supplementary Table S1). The average distance between adjacent markers was 0.72 cM. The largest LG was Gm18, with 339 SLAF markers and a length of 187.47 cM. The smallest LG was Gm04, with 260 SLAF markers and a length of 94.18 cM. The mean chromosome length was 126.72 cM (Table 2). A relatively high collinearity was observed between the 20 LGs and the reference genome (Supplementary Fig. S1), making the annotation of genes within QTL intervals feasible.

**Table 2:** Description of characteristics of the 20 linkage groups (LGs) in the soybean genetic map

LG^a^	No. of SLAFs	Distance (cM)	Average distance between markers (cM)	Largest gap (cM)
Gm01	228	130.35	0.57	5.27
Gm02	154	140.93	0.92	13.74
Gm03	231	147.28	0.64	15.40
Gm04	260	94.18	0.36	5.95
Gm05	86	99.60	1.17	8.20
Gm06	196	143.70	0.74	7.10
Gm07	142	118.42	0.84	14.89
Gm08	152	137.60	0.91	4.37
Gm09	147	151.62	1.04	13.95
Gm10	167	110.81	0.67	5.75
Gm11	69	112.48	1.65	8.71
Gm12	100	96.85	0.98	8.86
Gm13	178	176.98	1.00	7.02
Gm14	209	136.25	0.66	12.63
Gm15	237	99.96	0.42	6.97
Gm16	172	110.83	0.65	8.10
Gm17	217	120.51	0.56	4.82
Gm18	339	187.47	0.55	6.32
Gm19	161	120.24	0.75	7.18
Gm20	96	98.36	1.04	7.38
Maximum	339	187.47	1.65	15.40
Minimum	69	94.18	0.36	4.37
Total	3541	2534.42	0.72	–

^a^Linkage group

### QTL for fatty acid composition

We mapped 316 QTL to 20 soybean chromosomes for the five fatty acids. Of these QTL, 26 were identified for fatty acids across multiple environments, 64 for multiple fatty acids in single environments, and 226 for fatty acids in single environments (Supplementary Table S2). We focused mainly on the 26 stable QTL across multiple environments; these QTL were mapped to all chromosomes except Gm10 and Gm11 (Fig. 2), and the average phenotypic variance explained by individual QTL varied from 0.4 to 37.0% (Table 3). Eight of the 26 QTL explained the high phenotypic variance (>10%) for specific fatty acids. We detected three genes involved in fatty acid biosynthesis, and 66 genes encoding lipid-related transcription factors within the 26 stable QTL intervals (Table 3 and Supplementary Table S3).

**Table 3:** The stable QTL for specific fatty acids in soybean seed across multiple environments

QTL^a^	Trait^b^	Environment^c^	Chr^d^	Marker interval	LOD	PVE^e^ (%)	Novelty	Reported QTL or QTN	Gene annotation^f^
*qPA4_1*	PA	2009/2011	4	Gm04_47098–Gm04_44762	5.1–16.3	1.7–7.7	Novel		
*qPA6_1*		2009/2010	6	Gm06_8091–Gm06_2457	8.0–14.4	4.1–6.1	Novel		
*qPA8_1*		2010/2011	8	Gm08_9171–Gm08_38849	43.8–48.5	28.0–37.0		Bachlava et al. (2009)	
*qPA16_1*		2009/2010	16	Gm16_28849–Gm16_52098	5.9–13.5	2.2–7.5	Novel		
*qSA8_1*	SA	2009/2011	8	Gm08_53938–Gm08_3252	34.2–39.8	22.4–28.7		Li et al. (2015)	
*qSA12_1*		2009/2011	12	Gm12_1782–Gm12_30904	18.0–32.6	9.9–11.9	Novel		*GmFabG*
*qSA14_1*		2009/2010	14	Gm14_21994–Gm14_55450	3.8–28.8	3.0–7.0		Xie et al. (2012), Bachlava et al. (2009)	
*qSA18_1*		2009/2011	18	Gm18_84824–Gm18_77518	6.0–32.0	2.8–11.6		Fan et al. (2015)	
*qOA7_1*	OA	2010/2011	7	Gm07_58581–Gm07_29390	13.0–54.0	1.3–33.6		Fan et al. (2015)	
*qOA8_1*		2009/2010	8	Gm08_13258–Gm08_43422	6.0–6.1	1.1–6.5		Bachlava et al. (2009)	
*qOA9_1*		2009/2010	9	Gm09_45996–Gm09_5152	6.9–23.1	1.3–14.9		Fan et al. (2015), Li et al. (2015), Xie et al. (2012), Reinprecht et al. (2006), Wang et al. (2012)	*GmACP*
*qOA13_1*		2009/2011	13	Gm13_25899–Gm13_9722	11.9–28.9	2.3–3.5		Fan et al. (2015), Wang et al. (2012)	
*qOA17_1*		2009/2010	17	Gm17_33278–Gm17_7573	8.9–15.0	1.5–1.7		Xie et al. (2012)	
*qOA18_1*		2009/2011	18	Gm18_79781–Gm18_53964	52.1–68.1	8.6–27.4	Novel		
*qFA19_1*		2009/2011	19	Gm19_67930–Gm19_42890	10.4–20.5	1.0–4.4	Novel		
*qFA19_2*		2009/2010	19	Gm19_58422–Gm19_16080	3.6–20.9	1.8–4.4		Fan et al. (2015)	
*qFA19_1*	LA	2009/2010	19	Gm19_67930–Gm19_42890	7.9–26.0	0.7–12.0	Novel		
*qFA19_2*		2009/2010	19	Gm19_58422–Gm19_16080	28.3–40.3	13.6–19.3		Fan et al. (2015)	
*qLA1_1*		2009/2011	1	Gm01_79467–Gm01_15147	6.7–25.9	3.0–4.4	Novel		
*qLA4_1*		2009/2010	4	Gm04_67180–Gm04_61504	7.6–27.4	2.7–4.8	Novel		
*qLA5_1*		2010/2011	5	Gm05_27463–Gm05_53236	3.9–4.1	0.4–1.3		Bachlava et al. (2009)	
*qLA8_1*		2009/2010/2011	8	Gm08_47647–Gm08_8866	7.9–33.6	1.3–16.9		Fan et al. (2015)	
*qLNA2_1*	LNA	2009/2011	2	Gm02_57581–Gm02_69905	6.2–9.1	2.5–3.6	Novel		
*qLNA3_1*		2010/2011	3	Gm03_40606–Gm03_10118	13.1–42.1	3.7–21.7		Fan et al. (2015)	*GmFAD8*
*qLNA15_1*		2009/2010	15	Gm15_4886–Gm15_16198	7.9–35.2	3.0–17.6		Li et al. (2015)	
*qLNA16_1*		2010/2011	16	Gm16_11545–Gm16_47706	8.6–9.9	2.8–3.0		Diers and Shoemaker (1992)	
*qLNA19_1*		2010/2011	19	Gm19_45448–Gm19_57039	48.3–50.1	28.2–32.3		Fan et al. (2015)	
*qLNA20_1*		2009/2010/2011	20	Gm20_19811–Gm20_41673	14.9–44.4	3.6–26.0		Fan et al. (2015)	

^a^The name of QTL, is a composite of the influenced trait: PA (palmitic acid), SA (stearic acid), OA (oleic acid), LA (linoleic acid), and LNA (linolenic acid), followed by the chromosome number. For QTL underlying multiple fatty acids across various environments, the name is designated as a composite of FA (fatty acid) followed by the chromosome number

^b^The five predominant fatty acids are designated as follows: palmitic acid (PA), stearic acid (SA), oleic acid (OA), linoleic acid (LA), and linolenic acid (LNA)

^c^The three environments are designated as follows: 2009, 2010, and 2011

^d^Chromosome

^e^PVE indicates the phenotypic variance explained by individual QTL

^f^Gene annotation shows the essential genes involved in fatty acid biosynthesis discovered within the additive QTL intervals

Particularly, four stable QTL for palmitic acid (*qPA4_1*, *qPA6_1*, *qPA8_1*, and *qPA16_1*) explained 1.7–37.0% of the phenotypic variance. *qPA8_1* explained an averaged 32.5% of the phenotypic variance for palmitic acid. For stearic acid, four stable QTL (*qSA8_1*, *qSA12_1*, *qSA14_1* and *qSA18_1*) accounted for 3.0–28.7% of the phenotypic variance. *qSA8_1* and *qSA12_1* explained averaged 25.6 and 10.9% of phenotypic variance, respectively, and a *3*-*oxoacyl*-*ACP reductase* gene (*GmFabG*, *Glyma.12G092900*) was found within the genomic region of *qSA12_1*. For oleic acid, eight stable QTL were detected including *qOA7_1*, *qOA8_1*, *qOA9_1*, *qOA13_1*, *qOA17_1*, *qOA18_1*, *qFA19_1*, and *qFA19_2*, explaining 1.3–33.6% of the phenotypic variance. *qOA18*-*1* explained 18.0% of the phenotypic variance on average. An *acyl carrier protein* gene (*GmACP*, *Glyma.09G060900*) was found within the genomic region of *qOA9_1*. For linoleic acid, six stable QTL were identified, including *qFA19_1, FA19_2*, *qLA1_1*, *qLA4_1*, *qLA5_1*, and *qLA8_1*, explaining 0.4–19.3% of the phenotypic variance. *qFA19*-*2* explained an averaged 16.4% of the phenotypic variance. For linolenic acid, six QTL (*qLNA2_1*, *qLNA3_1*, *qLNA15_1*, *qLNA16_1*, *qLNA19_1*, and *qLNA20_1*) explained 2.5–32.3% of the phenotypic variance. *qLNA3_1*, *qLNA15_1* and *qLNA19_1* explained on average 12.7, 11.2, and 30.3% of the phenotypic variance, respectively. A *ω*-*fatty acid desaturase* gene (*GmFAD8*, *Glyma.03G056700*) was found within the genomic region of *qLNA3_1* (Table 3). Moreover, *qFA19_1* and *qFA19_2* contributed to both oleic and linoleic acid composition across multiple environments, suggesting a pleiotropic effect for multiple fatty acids (Table 3).

### Epistatic effect on fatty acid composition

Five epistatic QTL were detected for individual fatty acids, explaining 3.4–10.4% of the phenotypic variance (Table 4). Interestingly, a locus at 0 cM on Gm06 had epistatic effects with both loci on Gm07 and Gm10 for stearic acid across two environments (2009 and 2011), indicating a relatively stable epistatic effect (Table 4).

**Table 4:** Epistatic QTL for fatty acid contents in soybean seeds

Trait^a^	Chr^b^1	P^c^1 (cM)	Marker interval 1	Chr2	P2 (cM)	Marker interval 2	LOD(E)^d^	PVE^e^ (%)	A1^f^ (%)	A2 ^g^ (%)	A1byA2 ^h^ (%)
PA_2011	10	100	Gm10_49818–Gm10_38521	19	65	Gm19_62762–Gm19_39258	5.3	10.4	0.0001	−0.0882	−0.2088
SA_2009	6	0	Gm06_27520–Gm06_53504	7	115	Gm07_85356–Gm07_21186	5.2	5.6	−0.0353	0.0005	−0.1149
SA_2009	8	75	Gm08_54434–Gm08_14077	12	15	Gm12_67387–Gm12_12747	5.1	4.7	−0.0001	0.0033	−0.1036
SA_2011	6	0	Gm06_27520–Gm06_53504	10	90	Gm10_74377–Gm10_25475	5.1	7.2	−0.0141	0.0001	−0.1768
LNA_2011	2	30	Gm02_14960–Gm02_48191	13	140	Gm13_52660–Gm13_55424	5.1	3.4	−0.0011	0.0373	0.1473

^a^The five predominant fatty acids are designated as follows: PA (palmitic acid), SA (stearic acid), OA (oleic acid), LA (linoleic acid), and LNA (linolenic acid); the three environments are three continuous years from 2009 to 2011

^b^Chromosome

^c^Position in linkage groups in centiMorgans

^d^LOD score of epistatic QTL

^e^Phenotypic variance explained by epistatic QTL

^f^Additive effect of the first QTL

^g^Additive effect of the second QTL

^h^Epistatic effect between two QTL

## Discussion

SLAF-seq is an effective sequencing-based method for large-scale marker discovery and genotyping. Furthermore, SLAF-seq is a highly efficient approach for marker development that is relatively inexpensive and can be based on large populations (Li et al. 2014; Sun et al. 2013). This method has been widely used to construct high-density genetic maps and identify QTL for agronomic traits and disease resistance in various crops (Han et al. 2015; Qin et al. 2015; Su et al. 2016). However, the application of this method in QTL mapping for soybean fatty acid composition has not been reported. Therefore, we used SLAF-seq to construct a high-density genetic map and identify QTL for fatty acid composition.

### The effects of population size and marker density on QTL mapping

Efficiency and accuracy are important for QTL mapping (Li et al. 2010; Stange et al. 2013). We previously performed QTL mapping for fatty acid composition using a genetic linkage map based on 161 SSR marker sets and 100 RILs derived from the LHD2/NHZ cross (Fan et al. 2015). In the current study, all the 200 RILs from the same population were used to construct a new high-density genetic linkage map with 3541 SLAF markers. To analyze the efficiency and accuracy of QTL mapping with different population sizes and marker densities, we made a permutation for QTL detection of fatty acid composition using 100–200 RILs randomly selected from the population and compared the results each other and with our previous study. The number of detected QTL increased significantly with an increase in marker density and population size (Table 5). Therefore, the efficiency of QTL mapping improved significantly with an increase in population size and marker density. Many novel QTL could be identified with the improvement of QTL mapping.

**Table 5:** Number of QTL in soybean detected using different population sizes and marker densities

Population size	Marker density	Total QTL number
100	161	54
100	3541	149
150	3541	193
200	3541	316

On the other hand, almost all of QTL intervals (45 of the 47 overlapping QTL intervals) were reduced significantly with the increase of marker densities. In fact, 91% of QTL intervals were smaller than 5.0 cM, and 38% of QTL intervals were smaller than 1.0 cM. For instance, *qFA8_1* was mapped in an interval of 30.6 cM in our previous study (Fan et al. 2015), whereas the QTL interval was reduced to 1.1 cM in the current study (Supplementary Table S2). The smaller genomic region, in combination with the high collinearity of the genetic map with the reference genome sequence (Supplementary Fig. S1), will facilitate fine mapping of these QTL. That will help uncover the complex networks that govern fatty acid formation and regulation in soybean seeds. Another example, *qLNA19_1*, explaining an averaged 30.3% of the phenotypic variance for linolenic acid across two environments (2010 and 2011), was identified within a 0.5 cM interval, corresponding to a 55 kb genomic region in the soybean reference genome (Wm82.a2.v1). Therefore, the candidate genes within this region could be identified. Additionally, due to the low density of our previous genetic map, several closely linked QTL were assumed to be a single QTL. When the marker density and population size increased, these QTL could be detected accurately as several individual QTL. For instance, *qOA7_2* was mapped in a 17.9 cM interval in our previous study (Fan et al. 2015), whereas two adjacent additive QTL (*qFA7_7* and *qFA7_8*) were detected in the current study (Supplementary Table S2). Therefore, the accuracy of QTL mapping was improved significantly due to the increased population size and marker density.

### Comparison analysis revealed the novel stable QTL for fatty acids

According to the SoyBase database (http://soybase.org), hundreds of QTL have been detected for individual fatty acids (Alrefai et al. 1995; Bachlava et al. 2009; Brummer et al. 1997; Diers and Shoemaker 1992; Fan et al. 2015; Hyten et al. 2004; Panthee et al. 2006; Reinprecht et al. 2006; Wang et al. 2012, 2014; Xie et al. 2012). GWAS has also revealed 33 QTN associated with individual fatty acids (Li et al. 2015). We compared the QTL detected in our current study with the QTL and QTN reported previously according to their physical position in the soybean reference genome, and found that 108 of the total 316 QTL are novel (Supplementary Table S2). For the 26 stable QTL detected across multiple environments, nine are novel loci for individual fatty acids. Specifically, for saturated fatty acid, three novel QTL (*qPA4_1*, *qPA6_1*, and *qPA16_1*) were stably identified for palmitic acid, while one novel QTL, *qSA12_1*, was detected for stearic acid. For unsaturated fatty acid, two novel QTL (*qOA18_1* and *qFA19_1*) were detected for oleic acid; three novel QTL (*qFA19_1*, *qLA1_1* and *qLA4_1*) were identified for linoleic acid, while one novel QTL, *qLNA2_1*, contributed to linolenic acid (Table 3). These novel stable QTL could help elucidate the genetic basis of fatty acid accumulation and regulation.

### Major stable QTL and tightly linked markers could be applied for marker-assisted selection in soybean breeding programs

We also identified eight major stable QTL explaining the high phenotypic variance (>10%) for fatty acids. Particularly, *qPA8_1*, *qSA8_1*, *qLNA19_1* explained approximately one-third of the phenotypic variance for palmitic, stearic, and linolenic acids, respectively, across multiple environments. By contrast, our results suggested that the epistatic QTL had less effect on fatty acid contents compared with the additive effects of major QTL (Table 4). Therefore, these major QTL and tightly linked SLAF markers could be applied in MAS in soybean breeding programs.

### Gene annotation revealed fatty acid biosynthesis and lipid-related transcription factor genes

By annotating the genes within the 26 QTL intervals aginst Nr, Swiss-Prot, and KOG/COG databases, three essential genes involved in fatty acid biosynthesis (*GmACP*, *GmFabG*, and *GmFAD8*) were identified for oleic, stearic, and linolenic acids within the genomic region of *qOA9_1*, *qSA12_1*, and *qLNA3_1*, respectively. GmACP is an essential substrate that functions in upstream of *de novo* fatty acid biosynthesis. GmFabG is one of the core enzymes of fatty acid synthase (FAS). It catalyzes the reduction of acetoacetyl-ACP to β-hydroxybutyryl-ACP. In combination with other enzymes of FAS, palmitoyl-ACP (16:0-ACP) is produced (Somerville and Browse 1996). GmFAD8 is a ω-fatty acid desaturase that catalyzes the conversion of linoleic acid (18:2) to linolenic acid (18:3), and thereby is important for linolenic acid accumulation (Somerville and Browse 1996). Consistent with their functions, the QTL harboring these three genes contributed to oleic, stearic, and linolenic acids, respectively. The presence of these genes within the QTL suggests that they may contribute to the major effects of these loci.

In addition to the structural genes involved in fatty acid biosynthesis, 66 genes encoding lipid-related transcription factors, such as *MYB*, *WRKY*, *bZIP*, and *bHLH*, were also detected within the 26 QTL intervals (Supplementary Table S3). Several transcription factors play essential regulatory roles in fatty acid formation (Baud et al. 2007, 2009; Mendes et al. 2013; Mu et al. 2008; Raffaele et al. 2008; To et al. 2012; Wang et al. 2007). Therefore, the functional validation of these 66 genes in fatty acid regulation will help uncover the complex network underlying fatty acid composition in soybean seed.

In summary, we developed a high-density genetic map comprising 3541 SLAF markers using 200 RILs. With this high-resolution genetic map, we identified 26 stable QTL for fatty acid composition. Nine of these QTL are novel loci for individual fatty acids. Three genes involved in fatty acid biosynthesis (*GmACP*, *GmFabG*, and *GmFAD8*) were found within the genomic region of *qOA9_1*, *qSA12_1*, and *qLNA3_1*, respectively, suggesting they may contribute to the major effect for oleic, stearic, and linolenic acids, respectively. The stable and novel QTL detected in the present study will not only facilitate studies of the genetic basis of fatty acid formation and regulation, but they may also be useful in MAS for the improvement of soybean quality.

#### **Author contribution statement**

BL conducted the data analysis, QTL mapping, genomic comparative analysis, and wrote the manuscript. SXF, FKY, and YC extracted the DNA from the RIL populations, performed SNP calling, and developed the genetic linkage map for soybean. SRZ designed and edited the figures. FXH, SRY, and LZW provided advice on experimental design and edited the manuscript. JMS designed, supervised, and financed the work and edited the manuscript. All authors read and approved of the final manuscript.

## Electronic supplementary material

Below is the link to the electronic supplementary material.

Supplementary material 1 (DOCX 142 kb)

Supplementary material 2 (XLSX 149 kb)

Supplementary material 3 (XLSX 58 kb)

Supplementary material 4 (DOCX 30 kb)
