# Supplementary material for: High-resolution mapping of QTL for fatty acid composition in soybean using specific-locus amplified fragment sequencing
Source: Theor Appl Genet. 2017 Apr 7;130(7):1467–79. doi: 10.1007/s00122-017-2902-8 (PMC5487593; doi:10.1007/s00122-017-2902-8)
Supplement: Supplementary file 1 — Supplementary material 1 (DOCX 142 kb) [file 122_2017_2902_MOESM1_ESM.docx]

**
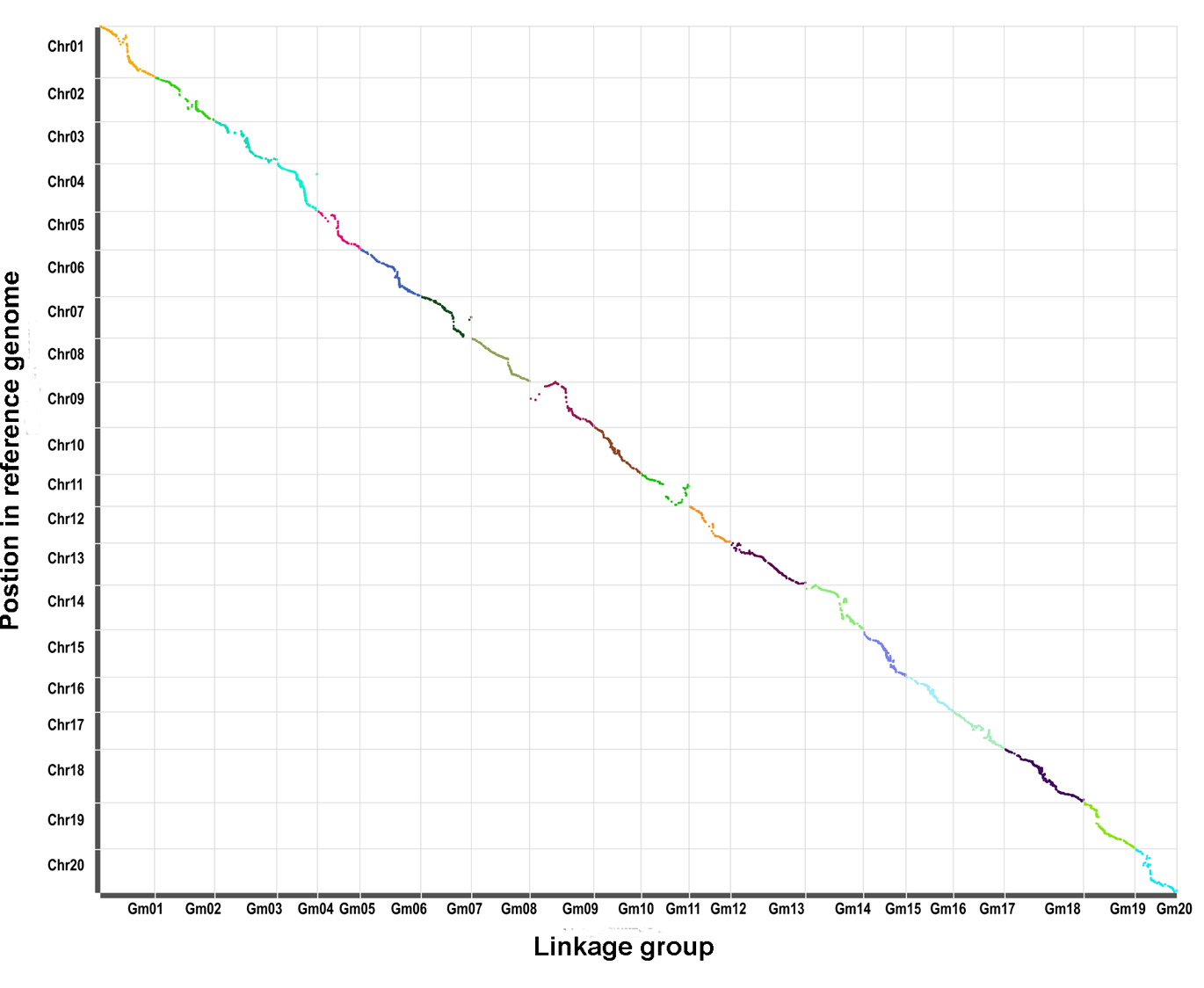
**

**Supplementary Fig. S1** Collinearity plot of 20 linkage groups with the soybean reference genome. The x-axis indicates the linear order of the linkage map and the y-axis indicates the linear order of the physical position in the soybean reference genome. The 3,541 SLAF markers were plotted as a scatter diagram. Different colors represent different chromosomes or linkage groups
