# Supplementary material for: High-resolution mapping of QTL for fatty acid composition in soybean using specific-locus amplified fragment sequencing
Source: Theor Appl Genet. 2017 Apr 7;130(7):1467–79. doi: 10.1007/s00122-017-2902-8 (PMC5487593; doi:10.1007/s00122-017-2902-8)
Supplement: Supplementary file 4 — Supplementary material 4 (DOCX 30 kb) [file 122_2017_2902_MOESM4_ESM.docx]

**Supplementary Table S3** Annotation genes encoding lipid-related transcription factors within the 26 stable QTL intervals

| Type | No. | QTL interval | Gene annotation |
| --- | --- | --- | --- |
| *MYB* | 1 | *qLA1_1* | *Lipid MYBJ6* |
|  | 2 | *qLNA2_1* | *Lipid MYB104-like* |
|  | 3 | *qLNA2_1* | *Lipid MYB83* |
|  | 4 | *qLNA3_1* | *LOC100801120* |
|  | 5 | *qLNA3_1* | *Trihelix lipid GT-3b-like* |
|  | 6 | *qPA6_1* | *Lipid MYB39-like* |
|  | 7 | *qPA6_1* | *MYB-related protein MYB4* |
|  | 8 | *qPA6_1* | *Lipid CPC* |
|  | 9 | *qSA8_1* | *Lipid MYB86-like* |
|  | 10 | *qSA8_1* | *Lipid APL-like* |
|  | 11 | *qLA8_1* | *Lipid RAX1-like* |
|  | 12 | *qOA9_1* | *Lipid MYB44-like* |
|  | 13 | *qOA9_1* | *MYB-related protein 306-like* |
|  | 14 | *qSA12_1* | *Lipid LHW-like* |
|  | 15 | *qOA13_1* | *Lipid MYB28-like* |
|  | 16 | *qLNA15_1* | *LOC100793978* |
|  | 17 | *qSA18_1* | *LOC100819797* |
| *WRKY* | 18 | *qLNA3_1* | *WRKY lipid 19-like* |
|  | 19 | *qLA4_1* | *WRKY lipid 40-1* |
|  | 20 | *qLA4_1* | *WRKY lipid 40-2* |
|  | 21 | *qLA5_1* | *WRKY lipid 19-like* |
|  | 22 | *qLA5_1* | *WRKY lipid 53-like* |
|  | 23 | *qPA6_1* | *WRKY lipid 31-like* |
|  | 24 | *qOA9_1* | *WRKY lipid 11* |
|  | 25 | *qOA9_1* | *WRKY lipid 19-like* |
|  | 26 | *qOA9_1* | *WRKY lipid 47-like* |
|  | 27 | *qSA12_1* | *WRKY lipid 31-like* |
| *bZIP* | 28 | *qPA4_1* | *Lipid bZIP125* |
|  | 29 | *qSA14_1* | *Lipid RF2a-like* |
|  | 30 | *qOA17_1* | *G-box-binding factor 1* |
| *bHLH* | 31 | *qPA8_1* | *Lipid bHLH71-like* |
|  | 32 | *qOA9_1* | *Lipid bHLH35-like* |
|  | 33 | *qLNA2_1* | *Lipid bHLH93-like* |
|  | 34 | *qLNA3_1* | *Lipid bHLH140-like* |
|  | 35 | *qPA4_1* | *Lipid bHLH63-like* |
|  | 36 | *qSA8_1* | *bHLH52-like-1* |
|  | 37 | *qSA8_1* | *bHLH52-like-2* |
|  | 38 | *qLA8_1* | *Lipid bHLH87-like* |
|  | 39 | *qOA9_1* | *Lipid bHLH041-like* |
| *Homeobox* | 40 | *qPA4_1* | *WUSCHEL-related homeobox 8-like* |
|  | 41 | *qOA9_1* | *Pathogenesis-related homeodomain protein-like* |
|  | 42 | *qPA6_1* | *Homeobox-leucine zipper protein HDG2-like* |
|  | 43 | *qLNA2_1* | *WUSCHEL-related homeobox 5-like* |
|  | 44 | *qOA9_1* | *Homeobox-leucine zipper protein HAT14-like* |
| *Zinc finger* | 45 | *qPA4_1* | *GATA lipid 5-like* |
|  | 46 | *qOA9_1* | *GATA lipid 21* |
|  | 47 | *qOA9_1* | *GATA lipid 26-like* |
|  | 48 | *qLA4_1* | *Zinc finger protein 3-like* |
|  | 49 | *qOA9_1* | *LSD1-like* |
| *AP2 domain* | 50 | *qLA4_1* | *PPLZ02-like* |
|  | 51 | *qLA5_1* | *Ethylene-responsive lipid CRF2-like* |
|  | 52 | *qPA6_1* | *Dehydration-responsive element-Binding protein 2C-like* |
|  | 53 | *qOA9_1* | *Ethylene-responsive lipid ERF017-like* |
|  | 54 | *qFA4_1* | *Ethylene-responsive lipid ERF016-like* |
| *NAC domain* | 55 | *qSA8_1* | *NAC domain-containing protein 74-like* |
|  | 56 | *qSA8_1* | *NAC domain-containing protein 42-like* |
| *Other* | 57 | *qPA6_1* | *Dr1-associated corepressor* |
|  | 58 | *qSA8_1* | *Nuclear lipid Y subunit C-2-like* |
|  | 59 | *qOA9_1* | *Lipid Y subunit A-3-like* |
|  | 60 | *qSA12_1* | *Glycine max lipid E2FB-like* |
|  | 61 | *qLNA20_1* | *Homeobox-leucine zipper protein HOX3-like* |
|  | 62 | *qPA4_1* | *Lipid GTE11* |
|  | 63 | *qSA8_1* | *Lipid JUNGBRUNNEN 1-like* |
|  | 64 | *qOA9_1* | *Brevis radix-like 4-like* |
|  | 65 | *qOA9_1* | *LOC102666574* |
|  | 66 | *qOA9_1* | *AATF-like (Apoptosis-antagonizing lipid)* |
